# Cyclic Ion Mobility Separations of Free Oxylipins

**DOI:** 10.1021/acs.analchem.6c03450

**Published:** 2026-09-16

**Authors:** Eric C. Gier, Facundo M. Fernández

**Affiliations:** † School of Chemistry and Biochemistry, 1372 Georgia Institute of Technology, Atlanta, Georgia 30332, United States; ‡ Parker H. Petit Institute for Bioengineering and Bioscience, Atlanta, Georgia 30332, United States

## Abstract

Oxylipins are oxygenated lipid metabolites derived from polyunsaturated fatty acids (PUFAs). They play interconnected roles in physiological processes such as inflammation and vascular function, and their dysregulation has been linked to diseases, including cardiovascular, metabolic, and immunological disorders. Liquid chromatography (LC) has a limited ability to resolve the large number of isomers and isobars within this diverse lipid class. Ion mobility spectrometry (IMS) offers an orthogonal gas-phase separation technique and has been shown to improve peak capacity for isomers that differ by more than 1–2% in collision cross section (CCS). High-resolution IMS platforms have progressively enabled the separation of isomers with increasingly similar gas-phase structures and offer the potential to separate complex mixtures in experiments such as mass spectrometry imaging where LC is not available. Here, we demonstrate the application of high-resolution cyclic ion mobility spectrometry–mass spectrometry (cIMS–MS) to separate and characterize six complex mixtures of authentic oxylipin standards. Results show that deprotonated oxylipin species often exhibit multiple mobility features that emerge only after extended separations, whereas metal-adducted species, especially sodium adducts, yield clearer single peaks. Optimized traveling wave parameters enabled the separation of mixtures containing three to five oxylipins within 3–10 m of effective path length before ion wrap-around occurred, resolving features with Δ^DT^CCS% of approximately 0.5% without front-end chromatographic separation. Multipass ^cIMS^CCS_N2_ measurements under optimized conditions were highly reproducible and closely matched the ^DT^CCS reference values from Moran-Garrido et al. and da Silva et al., with average deviations of 0.33–0.76%. These findings support the use of multipass cIMS experiments and ^cIMS^CCS measurements as valuable tools for oxylipin separation and annotation, particularly in the absence of chromatographic retention time data.

## Graphical abstract

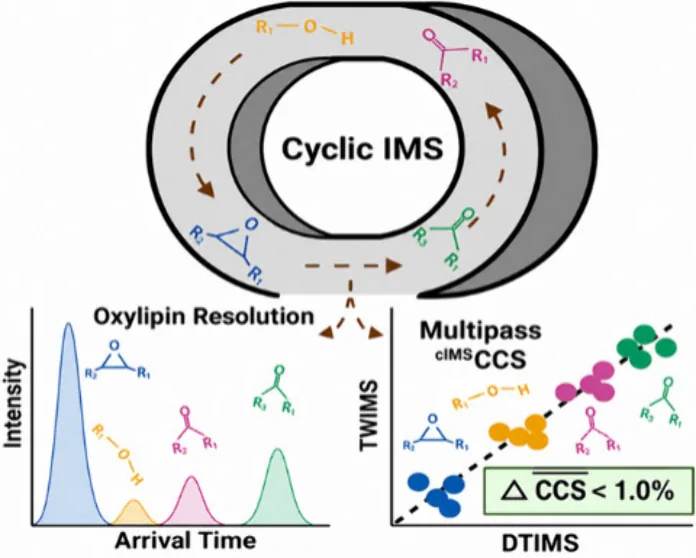

## Introduction

Oxylipins are a diverse group of bioactive lipids, characterized by numerous isomers, produced via enzymatic and radical-driven pathways from the oxidative metabolism of polyunsaturated fatty acids (PUFAs).^1^ Usually stored in esterified forms within larger lipid classes, oxylipins are released as free fatty acids to influence inflammation and vascular function; their dysregulation has been implicated in respiratory, cardiovascular, and neurodegenerative diseases.^2–5^ Free oxylipin isomeric complexity arises from enzymatic oxidation by cyclooxygenase (COX), lipoxygenase (LOX), and cytochrome P450 (CYP) enzymes, as well as autoxidation. This structural diversity manifests as linear and cyclized isomers bearing a variety of hydroxyl, epoxide, and ketone modifications, many of which share identical elemental composition and produce highly similar or indistinguishable tandem mass spectra, posing significant analytical challenges for confident identification.^6,7^

Reversed-phase liquid chromatography–mass spectrometry (LC–MS) is the primary method for oxylipin analysis because of its high sensitivity and selectivity, enabling detection of low-abundance species.^8^ Targeted LC–MS techniques match retention times and tandem mass spectrometry (MS/MS) fragmentation patterns to authentic standards, facilitating differentiation of many isomers and isobars and identification of coeluting analytes when they have distinct product ion spectra; however, this approach is often challenged by isomers that coelute and produce highly similar or indistinguishable MS/MS spectra.^8,9^ The sensitivity of multiple reaction monitoring (MRM) methods has recently enabled the direct detection of free oxylipins from tissue surfaces via mass spectrometry imaging (MSI) under ambient conditions.^10^ However, this method requires prior LC–MS/MS experiments, for example, on homogenized tissue, to establish which oxylipins are present and to inform MRM MSI transitions. When standards are unavailable, nontargeted LC–MS provides a comprehensive oxylipin profile, often using data-dependent acquisition to determine oxidation sites and types (such as epoxy, hydroxy, and keto groups), making it ideal for discovery studies.^11,12^ However, nontargeted approaches can be less sensitive, and limited precursor-ion selection resolution before MS/MS can make it difficult to distinguish structurally similar compounds.

Ion mobility spectrometry (IMS) quickly separates ions in the gas phase by size, shape, and charge, often within milliseconds. When paired with LC–MS, it boosts peak capacity.^13^ Drift tube IMS (DTIMS), the traditional variant, employs a uniform electric field to steer ions through a drift chamber containing neutral buffer gas, with no net flow.^14^ The mobility data obtained help identify compounds and are usually expressed as collision cross section (CCS) values, indicating an ion’s effective size and shape in the gas phase.^15–17^ Under DTIMS conditions, the time for an ion to reach its natural drift velocity is minimal, permitting CCS calculation via the Mason–Schamp equation (^DT^CCS).^18^ Variations in functional groups, their positions, adduct profiles, and double bonds influence the gas-phase size and shape of oxylipins, thereby impacting their mobility and enabling many isomers to be distinguished.^13,19^ Derivatization strategies such as the Paternò–Büchi reaction, which promotes cycloaddition across a carbon–carbon double bond, can alter lipid CCS so they fall outside the densely populated lipid region.^20^ Metal adducts generally increase ΔCCS% between oxylipin isomers compared to protonated or deprotonated forms, thus enhancing resolution.^21,22^ Studies integrating LC with DTIMS–MS show that very few free oxylipin isomers are completely unresolved across all adducts, underscoring the importance of comprehensive ^DT^CCS libraries for compound annotation.^22^ Nevertheless, many metal-adducted oxylipins still have ΔCCS% values below 2%, and methods such as MSI, which lack front-end separation, require high-resolution IM–MS techniques to distinguish isomers.

The popularization of IMS has led to the development of many high-resolution platforms with resolving powers reaching hundreds.^23^ Among these, traveling-wave (TW) IMS techniques, like structures for lossless ion manipulation (SLIM) and cyclic TWIMS (cIMS), achieve higher resolving power by extending the ion path.^24,25^ In TWIMS, a traveling electric field propels ions, and separation occurs because of differences in rollover frequency. cIMS allows ions to pass multiple times through a 1-meter separation cell because there is no net electric potential drop from start to end, and it has been used to separate and identify fatty acyl and other lipid isomers.^25,26^ Unlike DTIMS, CCS values in cIMS (^cIMS^CCS) are not directly calculable; instead, they are calibrated against ^DT^CCS, often using power-law relationships.^14,27^ CCS is crucial for oxylipin annotation when retention time data are missing, and MS/MS spectra are very similar or identical. Although most ^cIMS^CCS calibration techniques are designed for single-pass experiments, accurate multipass ^cIMS^CCS measurements are vital for analyzing complex mixtures of closely related oxylipins that cannot be separated in a single pass. Our tool, Multipass CCS Refiner, applies perturbation-corrected periodic drift times via the Lin and Costello method, streamlining CCS calibration in multipass cIMS by correcting for charge, reduced mass, and mass-dependent flight-time effects, as suggested by Ruotolo *et al*.^27–29^

This study examines the ion mobility properties of 23 free oxylipins (10 cyclized and 13 linear) grouped into 6 isomeric mixtures, using multipass cIMS in both positive and negative ion modes. By optimizing TW conditions and employing tandem cIMS (cIMS/cIMS), we resolve mixtures containing 3–5 oxylipin isomers. TW conditions of 375 m/s velocity and 18 V wave height, with 60-70 ms separation times, served as a practical starting point for oxylipin mixtures to separate isomers with similar gas-phase structures while limiting ion wrap-around. We measured multipass ^cIMS^CCS_N2_ values across four TW conditions to assess reproducibility and validated them against a DTIMS library and the literature.^21,22^ The Multipass CCS Refiner reduced the time required for post-processing CCS calibration curve creation and for studying unknown samples, enabling a workflow that completes within minutes.

## Materials and Methods

### Chemicals

Oxylipin standards (Table 1 and Figure S1) were obtained from Cayman Chemical (Ann Arbor, MI, USA). Sodium, lithium, and potassium acetate salts were purchased from Sigma-Aldrich (St. Louis, MO, USA). Optima-grade ACN and H_2_O were obtained from Fisher Scientific (Pittsburgh, PA, USA). Sodium formate and Major Mix calibration solutions were purchased from Waters Corporation (Wilmslow, UK). The SpheriCal IM–MS Low Range calibration mixture was obtained from Polymer Factory (Stockholm, Sweden).

**Table 1:** Elemental Composition, Neutral Mass, Compound Abbreviations, FA Shorthand Notation, Structural Category, and Isomerism Summary for the 23 Oxylipins Investigated, Grouped by Isomeric Mixture

mixture # (composition)	neutral mass (Da)	compound abbreviation^a^	structural category	isomerism summary
1	354.2406	PGD_1_	cyclized	constitutional isomers
FA 20:3;O3(C_20_H_3__4_O_5_)		PGE_1_		PGF_2_ diastereomers
		PGF_2α_		
		PGF_2β_		

2	320.2351	14*R*,15*S*-EpETrE	linear	constitutional isomers
FA 20:4;O(C_20_H_32_O_3_)		15(*R*)-HETE		15-HETE enantiomers
		15(*S*)-HETE		
		5(*S*)-HETE		
		(±)-8,9-EpETrE		

3	336.2301	5(*S*),12(*S*)-DiHETE	linear and cyclized	constitutional isomers
FA 20:4;O2(C_20_H_32_O_4_)		6-*trans*-LTB_4_		6-*trans*-LTB_4_ and 5(*S*),12(*S*)-DiHETE diastereomers
		8(*S*),15(S)-DiHETE		
		PGA_1_		

4	352.2250	LXA_4_	linear and cyclized	constitutional isomers
FA 20:4;O3(C_20_H_32_O_5_)		PGD_2_		
		PGE_2_		

5	318.2195	14(15)-EpETE	linear	constitutional isomers
FA 20:5;O(C_20_H_30_O_3_)		15(*S*)-HEPE		
		15-oxo-ETE		
		5-oxo-ETE		

6	334.2144	PGA_2_	cyclized	constitutional isomers
FA 20:5;O2(C_20_H_30_O_4_)		PGB_2_		
		PGJ_2_		

a Oxylipin abbreviations are detailed in the Supporting Information (Table S1).

### Sample Preparation

Oxylipin standards were grouped into 6 isomeric mixtures (Table S1), with full structural descriptions provided in the Supporting Information. Standards were prepared as individual compounds and as isomeric mixtures at 500 nM. Standards were initially dissolved in 100% H_2_O and then serially diluted in H_2_O/ACN (50:50, v/v). Acetate salt solutions (10 mM) of lithium, potassium, and sodium were prepared in H_2_O/ACN (50:50, v/v) and further diluted to a final sample concentration of 1 mM. Each standard was analyzed both individually and as part of its isomeric mixture.

### cIMS–MS Analysis

IMS separations were performed on a cIMS–mass spectrometry (cIMS–MS) platform (Waters Corporation, Wilmslow, U.K.), a commercially available instrument based on the SYNAPT G2-Si platform, in which the conventional 25 cm colinear separation cell is replaced by a 98 cm cyclic mobility cell positioned orthogonally to the primary ion path and operated with pulsed DC traveling waves.^25^ Electrospray ionization (ESI) was conducted in both positive and negative ion modes at applied voltages of 3 kV and 2 kV, respectively, and at a flow rate of 2 μL/min. Instrument parameters were tuned to preserve labile species, including a cone voltage of 20 V, a desolvation gas temperature of 200 °C and a flow of 600 L/h, a source offset of 10 V, a step-wave gradient of 10 V, trap and transfer cell collision energies of 5 and 3 V, respectively, and a transfer radio frequency of 150 V. Precursor ions were isolated in the quadrupole prior to separation, and N_2_, maintained at a pressure of 1.79 mbar, was used as the buffer gas throughout all experiments.

TW heights and velocities were optimized independently for each isomeric mixture and adduct type (Table S2). TW heights were first scanned to identify conditions that increased arrival-time differences between isomeric species. TW velocities were then adjusted to shift relative ion positions within the wave and further improve separation. Optimization was performed over TW heights of 15–30 V and velocities of 350–475 m/s, as higher wave energies often produced fragmentation within the separation cell, predominantly from water and CO_2_ losses.^30^ Ions underwent multiple passes through the cyclic mobility array, with pass counts determined manually for each separation. Separations were extended to the onset of ion wrap-around, where the fastest-moving ions complete an additional lap of the mobility cell and arrive at drift times comparable to the slowest-moving ion populations.^31^

### cIMS/cIMS–MS Analysis

cIMS/cIMS separations were performed by isolating specific mobility regions in the separation cell, storing them in the pre-store array, and reinjecting them for additional passes, while remaining mobility populations were transmitted directly to the time-of-flight detector without acquisition.^25,32^ Transmitting ions to the pre-store array remixes the population and separation is lost. Therefore, sliced ion populations must first undergo the same number of passes as were conducted in the cIMS experiment to return to the same level of resolution prior to additional separation. The arrival time distribution (ATD) obtained from each reinjected mobility slice was adjusted during data analysis to account for remixing within the pre-store array.^33^ Following mobility separation, ion populations were ejected to the time-of-flight detector, operated in V-mode, and data were collected over 2 min using 3 pushes per bin over 200 bins.

### Data Processing

Raw data were processed using MassLynx v4.2 and Quartz software (Waters Inc., Wilmslow, U.K.). Extracted arrival times were exported as. xlsx files. ATDs of individual species (dashed traces) and mixtures (solid black traces) were overlaid, plotted, and normalized to unity using custom R scripts (v4.5.2). Mobility feature centroids were determined by Gaussian fitting with nonlinear least-squares regression implemented using the nls function. Two-peak resolution (R_pp_) for peaks within isomeric mixtures was calculated as the difference in drift times between peak centroids divided by the sum of the peak widths, multiplied by a factor of 1.18 derived from the half-maximum definition (2.355σ/2).^34,35^ Half-height separation was defined at R_pp_ = 0.83, while baseline separation was defined as R_pp_ > 1.5.^35^ R_pp_ values were calculated from mixtures using the major mobility peak of each isomer under TW conditions optimized for group separation (Table S2).

### Multipass CCS Calculations

The SpheriCal® IM–MS Low Range calibration mixture was analyzed over a mass range of 300–1200 *m*/*z* under four TW conditions spanning 15–20 V and 350–450 m/s. Multipass calibration curves were generated from perturbation-corrected periodic drift times (t_pp_) obtained from [M + Na]^+^ adducts, using separation times (t_s_) of 0, 2, 10, 20, 30, 32, 50, 75, 100, and 110 ms.^27^ Automated t_pp_ values were calculated from Multipass CCS Refiner using linear regressions of (t_n_ – t_s_ – t_0_)/*n* versus t_s_/n, where t_n_ represents the measured arrival time, t_0_ the bypass time, and n the number of passes through the mobility cell.^28^ ATDs exhibiting ion wrap-around were excluded from the calibration datasets.

Calibrant ^DT^CCS_N2_ values were converted to reduced CCS (Ω′) to account for ion charge state and reduced mass effects. Calculated t_pp_ values were further adjusted for mass-dependent flight time contributions using the instrument-specific enhanced duty cycle delay coefficient, yielding t_D_′.^29,36^ Calibration curves were generated by linear regressions of ln­(Ω′) versus ln­(t_D_′), consistent with the power-law relationship between drift time and CCS, and the resulting exponential factors were used to calculate calibrated ^cIMS^CCS_N2_ values.^37^

All 23 oxylipin standards were subsequently analyzed as [M + Na]^+^ ions under the same four sets of TW conditions used for calibration and on the same day.^38^ The t_pp_ values were determined in the same manner as for the calibrants and were used to calculate ^cIMS^CCS_N2_ values using the corresponding calibration curves. Multipass ^cIMS^CCS_N2_ values were evaluated for variability across TW conditions and compared with recently published ^DT^CCS_N2_ databases and, when absent, with literature values.^21,22^ ATD data for calibrants and oxylipin standards were downloaded in. xlsx format from the introduction tab of Multipass CCS Refiner under the *Using the Application* section at: https://ericgier.shinyapps.io/Multipass-CCS-Refiner/. Instructions for processing the data within the application can be found in the first tab of the SpheriCal calibration example file (“Start Here”), and a tutorial is available through YouTube: www.youtube.com/watch?v=AXrbocbQGY8&t=0s. Additional updates to the application since its release include import/export functionality for calibration files and built-in or user-supplied calibrant tables, reducing manual data entry. Current and planned updates are summarized in the Announcements section, and complete documentation is available at: https://github.com/facundof2016/Multipass-CCS-Refiner.

## Results and Discussion

Oxylipin standards were selected to represent various oxygen-containing functional groups, including diols, epoxides, hydroxyls, and ketones within linear or cyclized structures. This structural diversity encompasses distinct types of constitutional isomerism: positional isomers that differ in the site of a shared functional group (e.g., 5­(S)-HETE versus 15­(S)-HETE, which differ only in hydroxyl position, or 14R,15S-EpETrE versus (±)-8,9-EpETrE, which differ only in epoxide position), and functional group isomers that differ in the identity of the oxygen-containing modification itself at a given site (e.g., 15­(S)-HETE versus 14R,15S-EpETrE) (Table 1). Diastereomeric relationships are also represented within this panel: PGF_2α_ and PGF_2β_ share identical connectivity but differ in configuration at C9, while 6-trans-LTB_4_ and 5­(S),12­(S)-DiHETE differ in both a stereocenter and E/Z double-bond configuration. In biological samples, variations in the position and type of functional groups, along with double-bond geometry, create numerous regio- and stereoisomers that often require high resolution to differentiate.^39,40^ These structural differences affect gas-phase ion conformations and their CCS, allowing separation by IMS. Notably, metal adducts frequently exhibit ΔCCS% values exceeding 2%, thereby aiding isomer resolution.^19,41^ cIMS was used to analyze 6 mixtures containing three to five oxylipin isomers without chromatographic separation, demonstrating its potential for MSI workflows. Due to the low endogenous oxylipin levels, especially in tissue sections, efficient separations with short path lengths and minimal reliance on cIMS/cIMS are necessary to minimize losses.^42^ Separations of complex oxylipin mixtures were optimized for a panel of 23 eicosanoids, including 18 from arachidonic acid (AA), 3 from dihomo-γ-linolenic acid (DGLA), and 2 from eicosapentaenoic (EPA) (Table S1).

### Ionization Behavior

Oxylipins easily form deprotonated ions in ESI(−) because they contain acidic groups like carboxylic acids. These [M – H]^−^ ions are common in LC–MS workflows due to their high ionization efficiency.^12,43^ In IMS, oxylipins appear in a narrow CCS range, with many isomers showing ΔCCS% < 0.5%.^15,19^ Their fluid gas-phase structures cause broad mobility peaks, often limiting or preventing separation. Moran-Garrido *et al*. reported a 100% detection rate for 74 oxylipins as [M – H]^−^ ions, with only 10% of isomer pairs showing ΔCCS% > 4%.^22^ Significant ΔCCS% differences were mainly seen in FA 20:5;O species, such as those in mixture 1, where 5-oxo-ETE showed three different deprotonated forms, two of which were separated from other isomers and accounted for most of the resolved species. To test how [M – H]^−^ oxylipins behave under multipass cIMS, separations with drift paths ranging from 1 to 7 meters were tested.

All 23 oxylipins were detected as [M – H]^−^ ions. Single-pass separations yielded ATDs with minimal resolution, aligning with DTIMS results.^21,22^ Extending the separation path to 7 m uncovered two or more gas-phase conformations for 17 of the 23 species (Figure S2). TW conditions were fine-tuned between 350–400 m/s and 15–18 V to enhance separation while limiting fragmentation within the mobility cell. Despite multiple deprotonated forms, some species showed partial or baseline resolution before ion wrap-around occurred. For instance, the main mobility feature of PGD_1_ clearly separated from the dominant features of other isomers in the FA 20:3;O3 mixture (Figure S2A), though minor features overlapped. The dominant mobility features of 5­(*S*),12­(*S*)-DiHETE and 8­(*S*),15­(*S*)-DiHETE were also well resolved (Figure S2C), with minor overlaps. However, all species in the FA 20:4;O3 mixture showed a single dominant mobility feature, and LXA_4_ was clearly separated from PGD_2_ and PGE_2_ (Figure S2D). The separation of the FA 20:5;O mixture was limited to a 3 m path length because 5-oxo-ETE rapidly occupied the entire separation space, displaying at least three deprotonated forms. Two of these forms were resolved from other isomers, consistent with DTIMS observations (Figure S2E).^22^

In ESI­(+) experiments, only the FA 20:5;O mixture consistently formed [M + H]^+^ adducts across more than half of its isomers. Similar to deprotonated species, many cases exhibited minor mobility features before ion wrap-around. Water-loss in-source fragmentation products were common and, in some instances, highly abundant, often showing broad mobility distributions. Longer pathlength separations heightened distribution complexity but also uncovered additional mobility features that could shed light on structural differences among isomers.^30^

Oxylipins readily formed metal adducts in ESI­(+), with sodiated species observed for most standards. When sodium acetate was added, all 23 oxylipins appeared as [M + Na]^+^ adducts, and many also as sodium acetate clusters in ESI(−), though with lower abundances. [M + Li]^+^ and [M + K]^+^ adducts were observed for over 85% of standards when their respective salts were present. Metal adducts of free oxylipins formed narrow mobility peaks and typically appeared as a single dominant feature, even after extended (>10 m) separations. These properties prompted a focus on metal adducts, particularly [M + Na]^+^, to optimize separation. Their stable conformations, strong ionization, and large ΔCCS% allowed for more effective resolution.

### cIMS Behavior of Prostaglandins

Prostaglandins feature a characteristic cyclopentane ring, a C13–C14 double bond, and a hydroxyl group at C15. Metal adducts with sodium, potassium, and lithium enable the differentiation of both constitutional isomers and stereoisomers.^21^ For instance, the larger gas-phase structure of [M + Na]^+^ of PGF_2α_ differs markedly from its isomers PGD_1_, PGE_1_, and the epimer PGF_2β_. Previous DTIMS studies showed Δ^DT^CCS% of about 3% between PGF_2α_ and PGE_1_, and about 4% between PGF_2α_ and PGD_1_.^21,22^ Meanwhile, PGF_2β_, being more compact, is only partially separated from PGE_1_, even with high-resolution demultiplexing (HRDm). Likewise, PGD_1_ and PGE_1_, which differ only by the position of the keto group at C11 versus C9, show limited separation as [M + Na]^+^ adducts in DTIMS.^22^

Previous studies examined these isomers in only three species, but this study investigated all four.^21,22^ Despite the added complexity, the FA 20:3;O3 mixture was clearly separated using cIMS after 8 meters of separation (Figure 1A). Before ion wrap-around, PGD_1_ and PGE_1_ nearly reached baseline resolution (R_pp_ = 1.48), and PGF_2β_ and PGE_1_ were fully separated (R_pp_ = 2.68). The most challenging pair was PGD_1_ and PGF_2β_ (R_pp_ = 1.07), but additional passes caused ion wrap-around, requiring cIMS/cIMS to further enhance resolution.

**Figure 1:**
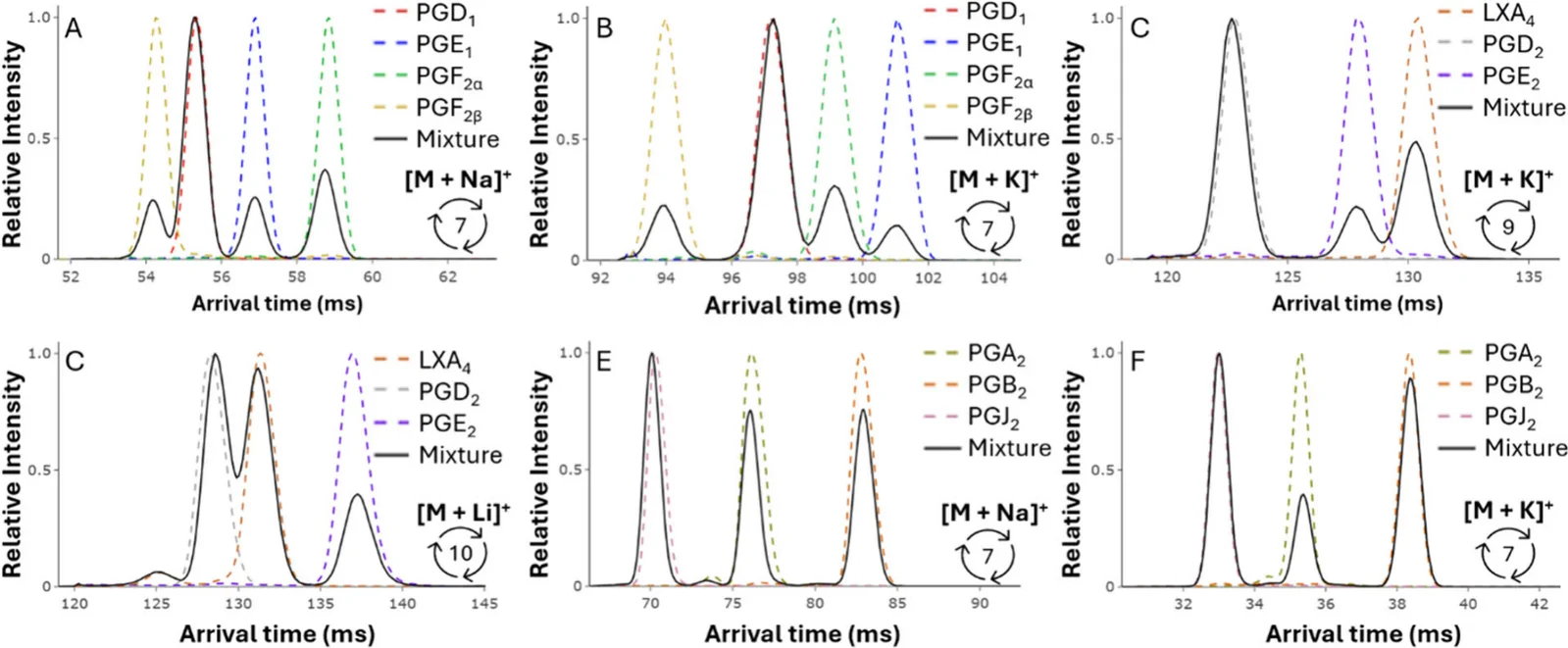
cIMS–MS separations of FA 20:3;O3 (mixture 1) as their [M + Na]^+^ (A) and [M + K]^+^ (B) adducts, FA 20:4;O3 (mixture 4) as their [M + K]^+^ (C) and [M + Li]^+^ (D) adducts, and FA 20:5;O2 (mixture 6) as their [M + Na]^+^ (E) and [M + K]^+^ (F) adducts, under individually optimized TW conditions (Table S2). Separations were extended to the onset of ion wrap-around. Solid black traces represent isomeric mixtures, and dashed traces correspond to individual isomer standards analyzed separately.

The separation of the FA 20:3;O3 isomeric mixture serves as a clear example of how adduct profile affects ion mobility and can enhance the resolution of isomeric pairs. Potassium adducts changed the relative mobilities of prostaglandin isomers, and after a 10-meter separation, they improved resolution between PGD_1_ and PGE_1_ (R_pp_ = 2.38), as well as between PGD_1_ and PGF_2β_ (R_pp_ = 2.12) (Figure 1B). Importantly, the migration order depended on the type of adduct: PGF_2α_, as lithium and sodium adducts, arrived last and was well separated from the other isomers. Conversely, with potassium adducts, PGF_2α_ arrived before PGE_1_, reducing the resolution between these species compared to sodium and lithium adducts (Figure S3A).

Adduct-dependent mobility was also observed for FA 20:4;O3 isomers. PGD_2_ and PGE_2_, which demonstrate nearly identical MS/MS fragmentation patterns and are usually separated by LC, were easily distinguished as sodium adducts (Figure S3B).^44^ However, adding the linear, LOX-derived LXA_4_ revealed a limitation of sodium adduct separations. LXA_4_ and PGE_2_ remained unresolved before ion wrap-around and showed minimal improvement even after an additional 40 m of separation, consistent with their small reported Δ^DT^CCS% (∼0.2%).^22^ Conversely, [M + K]^+^ (Figure 1C), [M + Li]^+^ (Figure 1D), and [M – H + CH_3_COONa]^−^ (Figure S3C) all improved resolution between LXA_4_ and PGE_2_, although these enhancements involved shifts in migration order and trade-offs in resolution with PGD_2_.

Cyclized dehydration products of PGE_2_ and PGD_2_, including PGA_2_, PGB_2_, and PGJ_2_, form a set of isomers that exhibit ion wrap-around in as little as 3 meters, with Δ^DT^CCS% values reaching up to 6.5%. While PGB_2_ and PGJ_2_ can be difficult to distinguish chromatographically, DTIMS has achieved their separation across various adduct profiles, and PGJ_2_ and PGA_2_ have also been nearly fully resolved by reversed-phase LC.^45^ In this study, all three isomers were examined together to evaluate mixture separation under conditions where ion wrap-around occurs rapidly. Lithium adducts showed significant separation between PGJ_2_ and PGB_2_ (R_pp_ = 6.02), but PGA_2_ and PGB_2_ were barely resolved (R_pp_ = 0.80) (Figure S3D). Although large ΔCCS% improves the separation of single species, it was, in this case, disadvantageous for the mixture, as it limited the separation window before wrap-around. More balanced CCS differences, such as those seen with sodium and potassium adducts, allowed baseline resolution of all three isomers within 3–4 meters (Figure 1E,F). While multiple mobility features were less common in metal adducts, PGA_2_ displayed a minor additional mobility feature in sodium, lithium, and potassium adducts, which in some cases was fully resolved in a few passes. In contrast, sodium acetate clusters needed more than 10 meters for similar resolution, and multiple mobility features were not detected for PGA_2_.

### cIMS Behavior of Linear Oxylipins

A similar correlation between structural lability and adduct profile was observed in linear oxylipins, including those in the FA 20:4;O and FA 20:5;O mixtures. Metal adduct formation again resulted in narrow mobility peaks, usually with a single dominant feature. DTIMS analyses of FA 20:5;O isomers revealed that [M + Na]^+^ of 14(15)-EpETE and 15-oxo-ETE are well separated; however, adding more isomers reduced the separation and complicated compound identification.^22^ Although higher mixture complexity also limited resolution in cIMS, a separation path length of 7 m was enough to distinguish 14(15)-EpETE from 15-oxo-ETE and also 15­(S)-HEPE from 5-oxo-ETE, which showed intermediate arrival times (Figure 2A). All four FA 20:5;O isomers were additionally separated as potassium and lithium adducts, with a minimum resolution of R_pp_ ≈ 1.1 between neighboring peaks before ion wrap-around (Figure 2B and Figure S3E).

**Figure 2:**
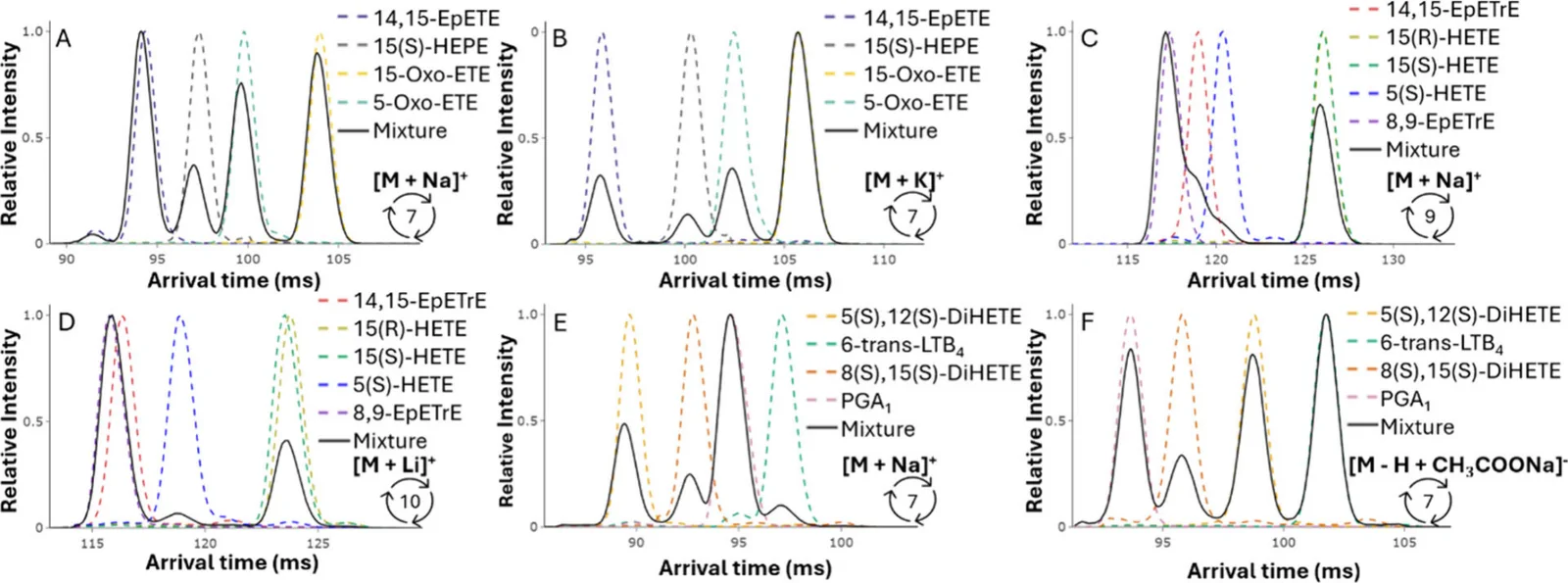
cIMS–MS separations of FA 20:5;O (mixture 5) as their [M + Na]^+^ (A) and [M + K]^+^ (B) adducts, FA 20:4;O (mixture 2) as their [M + Na]^+^ (C) and [M + Li]^+^ (D) adducts, and FA 20:4;O2 (mixture 3) as their [M + Na]^+^ adducts (E) and [M – H + CH_3_COONa]^−^ clusters (F), under individually optimized TW conditions (Table S2). Separations were extended to the onset of ion wrap-around. Solid black traces represent isomeric mixtures, and dashed traces correspond to individual isomer standards analyzed separately.

The FA 20:4;O mixture, which includes five linear oxylipins, proved more difficult to distinguish. da Silva *et al*. noted that a resolving power of approximately 180 is necessary to separate the constitutional isomers of this mixture as sodium acetate clusters.^21^ In cIMS, both 15-HETE enantiomers were partially resolved from epoxide-containing species beginning after 3–4 m of separation and optimized between 9–10 m as metal adducts in ESI­(+) (Figure 2C,D). Yet, 5-HETE showed intermediate arrival times, sometimes overlapping with nearby isomers depending on the adduct. Investigating sodium acetate clusters with cIMS revealed that the order of 5-HETE and the 15-HETE enantiomers shifted (Figure S3F) relative to sodium and lithium adducts, although both remained well separated from each other and from (±)-8,9-EpETrE. As expected, the 15­(R/S)-HETE enantiomers were not resolved under optimized TW conditions, even after extended runs of up to 40 m. Within this mixture, positional isomers such as 5-HETE and 15-HETE are also readily distinguished by reversed-phase LC, similar to the EpETrE regioisomer pair, and only the 15-HETE enantiomers remain challenging to resolve by either technique.^46^ Among these linear oxylipins, epoxide-containing species consistently had the shortest arrival times, consistent with previous findings by da Silva *et al*.^21^ However, the relative arrangement and separation of isomers depended heavily on factors such as adduct profile, functional groups, and number of double bonds.

FA 20:4;O2 proved comparatively resistant to full resolution, likely reflecting the close structural similarity among 5­(S),12­(S)-DiHETE, 6-*trans*-LTB_4_, and 8­(S),15­(S)-DiHETE, which share the same diol substitution pattern and differ only in hydroxyl position and double-bond geometry. Linear FA 20:4;O2 oxylipin isomers were clearly separated after 7 m as sodium adducts and sodium acetate clusters. Adding PGA_1_ reduced group separation in sodium adducts and positioned itself between 6-trans-LTB_4_ and 5­(*S*),12­(*S*)-DiHETE. In ESI(−), PGA_1_ sodium acetate clusters formed a more compact, early-moving peak, decreasing the number of passes before ion wrap-around (Figure 2E,F). These species also showed poor resolution as potassium adducts, and 6-trans-LTB_4_ exhibited low ionization with lithium.

No single adduct consistently maximized resolution; sodium and potassium adducts generally offered the most balanced compromise between separation and ion wrap-around, while lithium adducts, despite often producing the largest ΔCCS%, more frequently induced premature wrap-around in complex mixtures. Across all tested adduct types and TW conditions, 65.3% of isomer pair measurements achieved baseline resolution, 22.1% were partially resolved, and 12.6% remained unresolved (Table S2). Within standard (single-pass) cIMS, only the enantiomeric 15­(*R*)/15­(*S*)-HETE pair and the positional epoxide isomers 14R,15S-EpETrE and (±)-8,9-EpETrE remained unresolved across all tested adducts. Isomers that are unresolved or only partially resolved by cIMS alone may benefit from LC–cIMS workflows; the EpETrE pair, for example, is routinely resolved by conventional reversed-phase LC via retention time, reducing reliance on extended path lengths and extensively optimized TW conditions. For LC-free workflows, particularly in complex mixtures of four or more oxylipin isomers, cIMS/cIMS experiments can instead be used to improve resolution.

### cIMS/cIMS

Sodium adducts were chosen for cIMS/cIMS experiments because they generally produced single, distinct mobility features, showed efficient ionization, and exhibited little signal loss over long path lengths. Partially separated regions from multipass runs were isolated within the separation cell, transferred to the pre-store array, and reinjected for more passes. The regions, marked by vertical dashed boundaries in the ATDs, represent the mobility windows subjected to extended path lengths. Final reconstructed ATDs for each population were obtained by combining corrected, isolated portions of the ATD collected from individual reinjection events, after correcting for remixing within the pre-store array (see Figure S4).^33^ Regions outside the dashed boundaries were ejected directly to the detector without collection. While this method might be less effective for low-abundance species, cIMS/cIMS enhances the resolution of complex mixtures that would otherwise be limited by ion wrap-around in continuous cIMS experiments.

cIMS/cIMS was applied to mixtures of FA 20:3;O3, FA 20:4;O, and FA 20:4;O2 isomers (Figure 3 and Figure S5). For FA 20:3;O3, a total pathlength of 18 m was required to resolve PGD_1_ and PGF_2__β_, which exhibit a small Δ^DT^CCS of ∼0.57% (Figure 3A,B). Similarly, for FA 20:4;O2, a total pathlength of 17 m enabled resolution of the closely spaced three-component region comprising PGA_1_, 6-trans-LTB_4_, and 8­(S),15­(S)-DiHETE (Figure S5), where neighboring species differed in ^DT^CCS by ∼0.9–1.2%.

**Figure 3:**
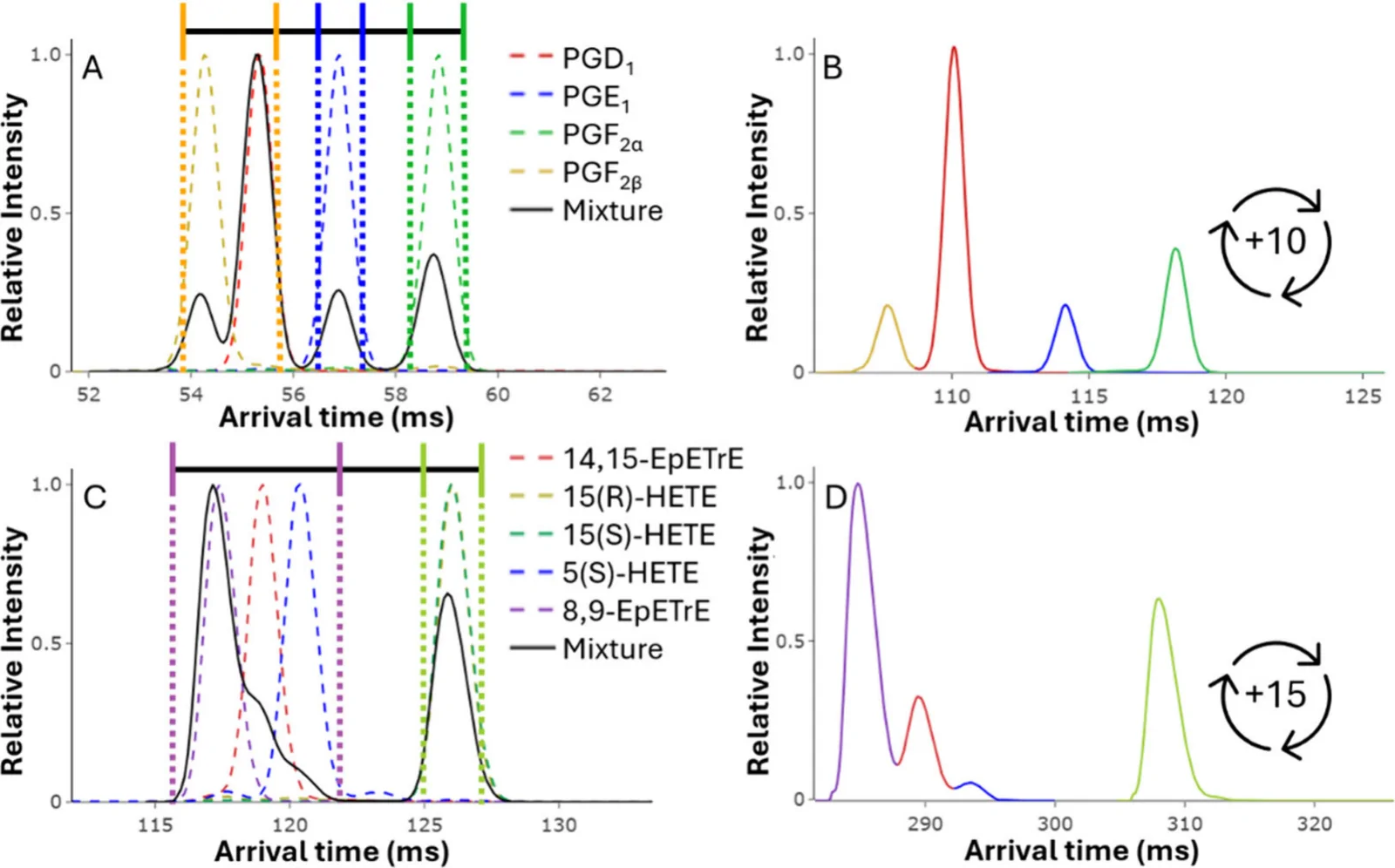
Multipass cIMS–MS and cIMS/cIMS–MS separations of FA 20:3;O3 (mixture 1) and FA 20:4;O (mixture 2) isomers as their [M + Na]^+^ adducts extended to the onset of ion wrap-around. The 8 m separation of FA 20:3;O3 was divided into three mobility slices defined by vertical boundaries (A), each extended by an additional 10 m, yielding a reconstructed ATD with a total pathlength of 18 m (B). The 9 m separation of FA 20:4;O was divided into two slices (C), each extended by 15 m to a total cIMS pathlength of 24 m (D). Solid traces represent mixtures; dashed traces represent individual standards.

The FA 20:4;O mixture, containing five isomers, was subjected to a combined 24 m of separation until the second onset of ion wrap-around in the three-component region highlighted in purple (Figure 3C,D). Although an additional 15 m of pathlength improved separation relative to cIMS experiments alone, complete resolution was not achieved. These observations are consistent with the small Δ^DT^CCS% between adjacent 14*R*,15*S*-EpETrE and 5-HETE (∼0.49%) and between (±)-8,9-EpETrE and 14*R*,15*S*-EpETrE (∼0.56%), as well as the near co-mobility of 15­(*R*)- and 15­(*S*)-HETE. Together, these results demonstrated that cIMS/cIMS experiments can substantially improve resolution in complex oxylipin mixtures while highlighting limitations for isomers with minor differences in gas-phase structure.

### Multipass CCS Calculations

Mobility values determined from first-principles in DTIMS are considered a primary standard and serve as the basis for calculating ^DT^CCS values using the Mason–Schamp equation.^47^ These measurements are validated through rigorous quality control procedures and generally show excellent reproducibility, with intralaboratory RSD typically ≤0.1%.^15^ Moran-Garrido *et al*. conducted the largest interlaboratory comparison of oxylipin ^DT^CCS_N2_ values to date, evaluating oxylipins across five laboratories in both ionization modes. Average interlaboratory deviations were generally <1.5%, with maximum deviations up to 3%.^22^

In TWIMS, CCS calibration is typically performed by fitting power functions or polynomials to reference ^DT^CCS values for calibrant ions.^48^ Existing calibration strategies for ^cIMS^CCS have largely been developed for single-pass separations; however, complex oxylipin mixtures often remain unresolved after only 1 m of separation. As a result, accurate multipass ^cIMS^CCS values are highly desirable for future oxylipin studies in biological samples to improve annotation confidence. Extending ^cIMS^CCS calibration to multipass experiments can be both time- and labor-intensive, requiring the generation of multiple calibration curves in which analytes and calibrants undergo identical separation path lengths.

Modern calibration strategies leveraging average ion velocities and periodic drift times, the duration to complete a cycle through the separation region, have been shown to produce accurate ^cIMS^CCS values, usually within about 1% of ^DT^CCS reference data.^38,49^ In this study, a periodic drift time-based method, developed by Lin and Costello, was used to address perturbations in electric potential caused by field switching during ion ejection. It also provides a simple, practical framework for calculating multipass ^cIMS^CCS.^27^ Calibration was carried out using t_pp_ values, derived over various separation times (Figure S6A). Further corrections included adjusting CCS values for ion charge state and reduced mass, as well as refining t_pp_ values to account for mass-dependent flight-time variations (Figure S6B). Multipass ^cIMS^CCS_N2_ values for oxylipin standards were then derived from their respective t_D_′ measurements under the same TW conditions and compared to literature ^DT^CCS_N2_ values (Figure 4A–C and S6C). Selecting calibrant species carefully was critical to avoid multiple mobility features at longer path lengths, which can complicate Gaussian fitting.

**Figure 4:**
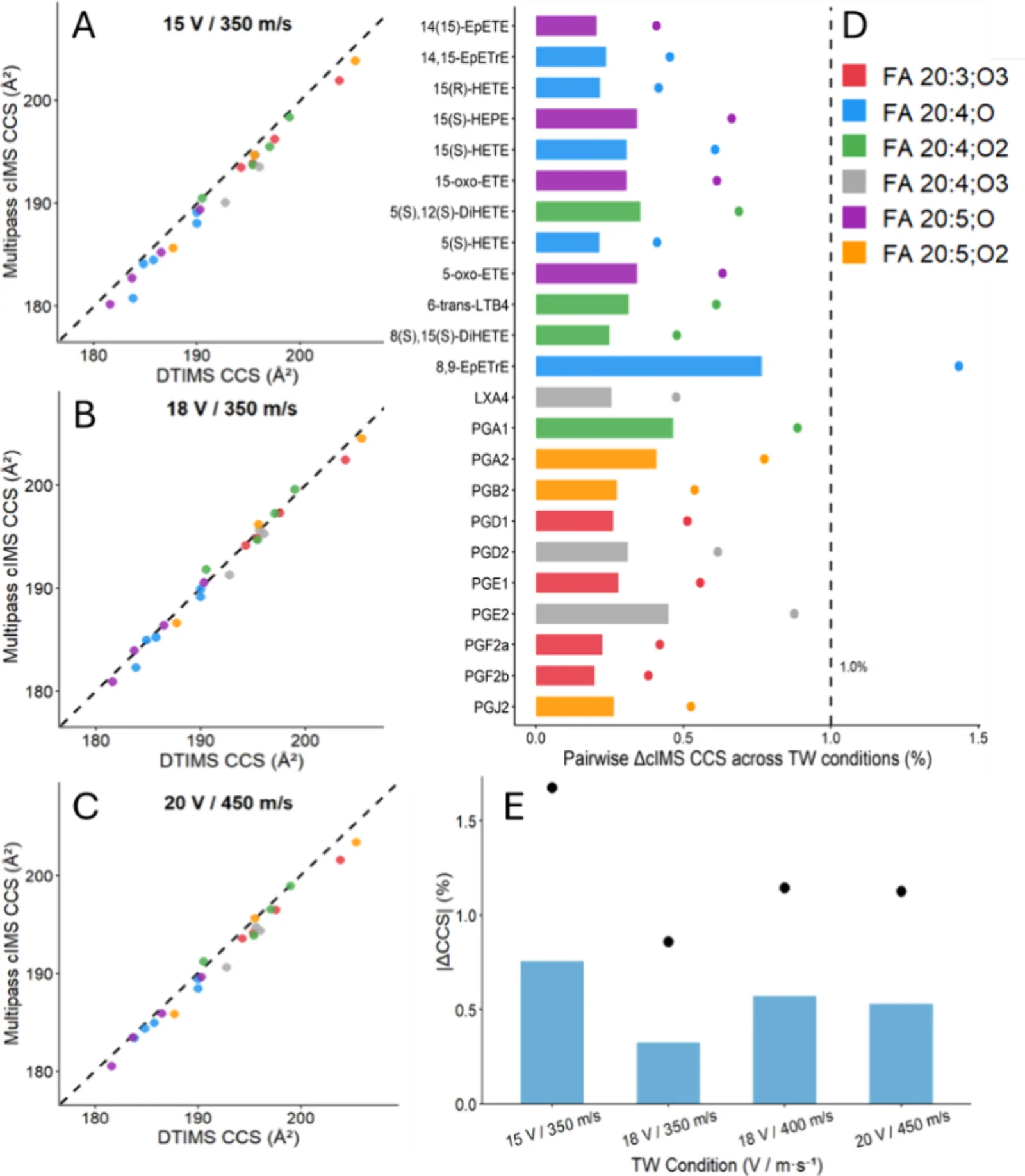
Comparison of calculated multipass ^cIMS^CCS_N2_ values with ^DT^CCS_N2_ reference values for [M + Na]^+^ adducts of oxylipins. Scatter plots are shown for three TW conditions optimized for oxylipins: 15 V/350 m/s, 18 V/350 m/s, and 20 V/450 m/s (A–C). The dashed line represents unity, and data points are colored by isomeric mixture. Variability in multipass ^cIMS^CCS_N2_ within each TW condition is shown, where horizontal bars indicate the average and dots indicate the maximum percent |ΔCCS| (D). The average and maximum percent |ΔCCS| values between multipass ^cIMS^CCS_N2_ and ^DT^CCS_N2_ are summarized in (E). The complete list of multipass ^cIMS^CCS_N2_ and ^DT^CCS_N2_ values are provided in the Supporting Information (Table S3).

Calibration curves were created across four TW conditions using Multipass CCS Refiner to assess variability in multipass ^cIMS^CCS_N2_ under conditions mimicking optimized separations for complex oxylipin mixtures (Figure S7).^28^ [M + Na]^+^ adducts were chosen for these experiments because they were common to all species, well-documented in existing libraries, and produced single mobility features. Pairwise comparison of [M + Na]^+^ oxylipin isomers across TW conditions showed average ^cIMS^CCS_N2_ differences ranging from 0.20% to 0.77%, with a maximum single measurement deviation of 1.44% for (±)-8,9-EpETrE (Figure 4D). Out of 92 measurements (23 analytes tested under four TW conditions), only 12 values differed by more than 1.0% from the ^DT^CCS_N2_ reference data, with overall average deviations between 0.33 and 0.76% (Figure 4E). This consistency aligns with known interlaboratory variability and supports the reliability of multipass ^cIMS^CCS for oxylipin analysis. Implementing calibration workflows applicable across various cIMS TW conditions and adaptable to cIMS/cIMS should further improve the analysis of complex oxylipin mixtures. Recent studies indicate that multipass ^cIMS/cIMS^CCS can be calculated by modifying t_0_ to account for all injection, separation, and storage steps outside the final separation, with platforms such as SLIM further validating TWIMS CCS values over longer path lengths.^48,50^

## Conclusions

This work demonstrates that adduct selection and TW parameter optimization are key variables for resolving complex oxylipin mixtures with cIMS, and that meaningful separations of isomeric species are achievable, even at short effective path lengths. Although annotation of oxylipins is challenging in the absence of retention time information, our findings support multipass ^cIMS^CCS as a reliable annotation metric to facilitate this task, which we believe will be particularly important for species such as PGE_2_ and PGD_2_, whose MS/MS fragmentation spectra are nearly identical. While minimizing path length mitigates ion losses inherent to extended cIMS separations, the sensitivity of cIMS–MS platforms may still be the ultimate limiting factor for trace-level analysis of oxylipins; enhanced ionization strategies, such as the addition of metal salts to the sprayed solvent, will likely be necessary for improving oxylipin detection and increasing coverage.

LC–cIMS-MS approaches offer complementary advantages in oxylipin studies, including reduced sample complexity, decreased ion suppression, and orthogonal separation, which will likely reduce the need for extensive TW optimization and extended cIMS/cIMS separations. Nevertheless, for complex samples analyzed without LC, longer separation path lengths, such as those achievable with cIMS/cIMS, will likely be necessary to fully resolve the oxylipin isomeric landscape. We anticipate that high-quality cIMS/cIMS CCS measurements will play an increasingly important role in oxylipin identification, enabling more confident annotation in both LC-coupled and LC-free workflows, ideally through methods that remain time-efficient and broadly accessible to the community.

## Supplementary Material



## Notes

Detailed chemical standard information, free oxylipin structures, cIMS–MS instrumental parameters, and separation path lengths for the workflows are provided. Additional ATD data for oxylipin separations are included for deprotonated and metal-adduct species, as individual and isomeric mixtures. Additional cIMS/cIMS experiments, including reconstruction procedures for extended separations, are presented. Calibration and multipass CCS determination workflow plots are included for regression analyses across multiple TW conditions. Calculated CCS values, R_pp_, and comparisons between multipass ^cIMS^CCS_N2_ and literature ^DT^CCS_N2_ values are also provided (PDF)
