# Supplementary material for: Cyclic Ion Mobility Separations of Free Oxylipins
Source: Anal Chem. 2026 Sep 16;98(38):28218–28. doi: 10.1021/acs.analchem.6c03450 (PMC13629299; doi:10.1021/acs.analchem.6c03450)
Supplement: Supplementary file 1 [file ac6c03450_si_001.pdf]

Supporting Information for

Cyclic Ion Mobility Separations of Free Oxylipins

Eric C. Gier[1], Facundo M. Fernández[1,2*]

[1]School of Chemistry and Biochemistry, Georgia Institute of Technology, Atlanta, GA 30332 (USA).

[2]Parker H. Petit Institute for Bioengineering and Bioscience, Atlanta, GA 30332 (USA).

*Corresponding author: Facundo Fernández (facundo.fernandez@chemistry.gatech.edu)

TABLE OF CONTENTS

Table S1. Summary of Standards S2

Table S2. cIMS: TW conditions, pass number, and R_{pp} values S4

Table S3. $^{DT}CCS_{N2}$ and multipass $^{cIMS}CCS_{N2}$ values S7

Figure S1. Chemical structures of oxylipin standards S8

Figure S2. ATDs of $[M–H]^-$ oxylipin isomers S9

Figure S3. Additional ATDs of oxylipin isomers as metal adducts S9

Figure S4. cIMS/cIMS ATD reconstruction workflow S10

Figure S5. cIMS/cIMS of FA 20:4;O2 S11

Figure S6. Multipass CCS calibration workflow (18 V, 400 m/s) S11

Figure S7. Multipass CCS calibration curves and linear fits across TW conditions S12

Table S1. Oxylipin standards were organized into six isomeric mixtures by sum composition. Mixture sum composition, molecular formula, precursor polyunsaturated fatty acid (PUFA), LIPID MAPS ID, systematic names and synonyms corresponding to each common name used in the main text are provided.

Mixture (Formula)	Common Name (LIPID MAPS ID)	Precursor PUFA* (Pathway)	Systematic Name	Synonyms
**FA 20:3;O3** ($C_{20}H_{34}O_5$)	**PGD₁** (LMFA03010049)	DGLA (COX)	9S,15S-dihydroxy-11-oxo-13E-prostaenoic acid	Prostaglandin D1
	**PGE₁** (LMFA03010134)	DGLA (COX)	9-oxo-11R,15S-dihydroxy-13E-prostaenoic acid	Prostaglandin E1
	**PGF$_{2\alpha}$** (LMFA03010002)	AA (COX)	9S,11R,15S-trihydroxy-5Z,13E-prostadienoic acid	Prostaglandin F2alpha<br>Prostaglandin F2a<br>Enzaprost<br>Dinoprost<br>Amoglandin<br>Cyclosin
	**PGF$_{2\beta}$** (LMFA03010025)	AA (COX)	9R,11R,15S-trihydroxy-5Z,13E-prostadienoic acid	Prostaglandin F2beta<br>Prostaglandin F2b
**FA 20:4;O** ($C_{20}H_{32}O_3$)	**14R,15S-EpETrE** (LMFA03080001)	AA (CYP)	14R,15S-epoxy-5Z,8Z,11Z-eicosatrienoic acid	14,15-EET
	**15(R)-HETE** (LMFA03060030)	AA (LOX/COX)	15R-hydroxy-5Z,8Z,11Z,13E-eicosatetraenoic acid	
	**15(S)-HETE** (LMFA03060001)	AA (LOX/COX)	15S-hydroxy-5Z,8Z,11Z,13E-eicosatetraenoic acid	
	**5(S)-HETE** (LMFA03060002)	AA (LOX)	5S-hydroxy-6E,8Z,11Z,14Z-eicosatetraenoic acid	5-HETE
	**(±)-8,9-EpETrE** (LMFA03080003)	AA (CYP)	8,9-epoxy-5Z,11Z,14Z-eicosatrienoic acid	8,9-EET
**FA 20:4;O2** ($C_{20}H_{32}O_4$)	**6-trans-LTB₄** (LMFA03020013)	AA (LOX)	5S,12R-dihydroxy-6E,8E,10E,14Z-eicosatetraenoic acid	6-trans-Leukotriene B4
	**5(S),12(S)-DiHETE** (LMFA03060101)	AA (LOX)	5S,12S-dihydroxy-6E,8Z,10E,14Z-eicosatetraenoic acid	
	**8(S),15(S)-DiHETE** (LMFA03060050)	AA (LOX)	8S,15S-dihydroxy-5Z,9E,11Z,13E-eicosatetraenoic acid	
	**PGA₁** (LMFA03010005)	DGLA (COX)	9-oxo-15S-hydroxy-10Z,13E-prostadienoic acid	Prostaglandin A1<br>15α-Hydroxy-9-oxo-10,13E-prostadienoic acid

**FA 20:4;O3** ($C_{20}H_{32}O_5$)	**LXA_4** (LMFA03040001)	AA (LOX)	5S,6R,15S-trihydroxy-7E,9E,11Z,13E-eicosatetraenoic acid	Lipoxin A4<br>5S,6R-Lipoxin A4
	**PGD_2** (LMFA03010004)	AA (COX)	9S,15S-dihydroxy-11-oxo-5Z,13E-prostadienoic acid	Prostaglandin D2<br>11-Dehydroprostaglandin F2-alpha
	**PGE_2** (LMFA03010003)	AA (COX)	9-oxo-11R,15S-dihydroxy-5Z,13E-prostadienoic acid	Prostaglandin E2<br>Dinoprostone<br>Prostin E2
**FA 20:5;O** ($C_{20}H_{30}O_3$)	**14(15)-EpETE** (LMFA03000003)	EPA (CYP)	(+/-)-14(15)-epoxy-5Z,8Z,11Z,17Z-eicosatetraenoic acid	
	**15(S)-HEPE** (LMFA03070009)	EPA (LOX)	15S-hydroxy-5Z,8Z,11Z,13E,17Z-eicosapentaenoic acid	
	**15-oxo-ETE** (LMFA03060051)	AA (LOX)	15-oxo-5Z,8Z,11Z,13E-eicosatetraenoic acid	
	**5-oxo-ETE** (LMFA03060011)	AA (LOX)	5-oxo-6E,8Z,11Z,14Z-eicosatetraenoic acid	
**FA 20:5;O2** ($C_{20}H_{30}O_4$)	**PGA_2** (LMFA03010035)	AA (COX)	9-oxo-15S-hydroxy-5Z,10Z,13E-prostatrienoic acid	Prostaglandin A2
	**PGB_2** (LMFA03010018)	AA (COX)	15S-hydroxy-9-oxo-5Z,8(12),13E-prostatrienoic acid	Prostaglandin B2
	**PGJ_2** (LMFA03010019)	AA (COX)	11-oxo-15S-hydroxy-5Z,9,13E-prostatrienoic acid	Prostaglandin J2<br>8-epi-15-J2t-IsoP

* Abbreviations correspond to PUFAs: arachidonic acid (AA), dihomo-γ-linolenic acid (DGLA), and eicosapentaenoic acid (EPA); and major enzymatic pathways: cyclooxygenase (COX), lipoxygenase (LOX), and cytochrome P450 (CYP).

Table S2. Summary of TW conditions and pairwise R_{pp} values for oxylipin isomer separations. All separations were extended to the onset of ion wrap-around, and R_{pp} values were calculated from isomeric mixtures of metal adducts.

Figure	Notation (Species Level)	Ion Profile	TW Conditions	Isomer Pair	R_{pp}
Figure 1A	FA 20:3;O3	$[M + Na]^+$	475 m/s, 30 V<br>8 passes	PGD_1 | PGE_1	1.48
				PGD_1 | $PGF_{2\alpha}$	3.10
				PGD_1 | $PGF_{2\beta}$	1.07
				PGE_1 | $PGF_{2\alpha}$	1.72
				PGE_1 | $PGF_{2\beta}$	2.68
				$PGF_{2\alpha}$ | $PGF_{2\beta}$	4.36
Figure 1B	FA 20:3;O3	$[M+K]^+$	475 m/s, 25 V<br>10 passes	PGD_1 | PGE_1	2.38
				PGD_1 | $PGF_{2\alpha}$	1.17
				PGD_1 | $PGF_{2\beta}$	2.12
				PGE_1 | $PGF_{2\alpha}$	1.18
				PGE_1 | $PGF_{2\beta}$	4.62
				$PGF_{2\alpha}$ | $PGF_{2\beta}$	3.33
Figure 1C	FA 20:4;O3	$[M+K]^+$	375 m/s, 18 V<br>8 passes	LXA_4 | PGD_2	3.19
				LXA_4 | PGE_2	1.03
				PGD_2 | PGE_2	2.33
Figure 1D	FA 20:4;O3	$[M+Li]^+$	400 m/s, 15 V<br>6 passes	LXA_4 | PGD_2	0.83
				LXA_4 | PGE_2	1.85
				PGD_2 | PGE_2	2.64
Figure 1E	FA 20:5;O2	$[M+Na]^+$	375 m/s, 16 V<br>4 passes	PGA_2 | PGB_2	3.23
				PGA_2 | PGJ_2	3.06
				PGB_2 | PGJ_2	6.25
Figure 1F	FA 20:5;O2	$[M+K]^+$	375 m/s, 22 V<br>3 passes	PGA_2 | PGB_2	3.04
				PGA_2 | PGJ_2	2.52
				PGB_2 | PGJ_2	5.45
Figure 2A	FA 20:5;O	$[M+Na]^+$	375 m/s, 16 V<br>7 passes	5-oxo-ETE | 14(15)-EpETE	2.46
				5-oxo-ETE | 15(S)-HEPE	1.18
				5-oxo-ETE | 15-oxo-ETE	1.81
				14(15)-EpETE | 15(S)-HEPE	1.32
				14(15)-EpETE | 15-oxo-ETE	4.30
				15(S)-HEPE | 15-oxo-ETE	3.04
Figure 2B	FA 20:5;O	$[M+K]^+$	400 m/s, 18 V<br>7 passes	5-oxo-ETE | 14(15)-EpETE	3.15
				5-oxo-ETE | 15(S)-HEPE	1.10
				5-oxo-ETE | 15-oxo-ETE	1.50
				14(15)-EpETE | 15(S)-HEPE	2.19
				14(15)-EpETE | 15-oxo-ETE	4.64
				15(S)-HEPE | 15-oxo-ETE	2.65

Figure 2C	FA 20:4;O	$[M+Na]^+$	400 m/s, 18 V<br>9 passes	14R,15S-EpETrE | 15(R)-HETE	2.46
				14R,15S-EpETrE | 15(S)-HETE	2.46
				14R,15S-EpETrE | 5(S)-HETE	unresolved
				14R,15S-EpETrE | (±)-8,9-EpETrE	unresolved
				15(R)-HETE | 15(S)-HETE	unresolved
				15(R)-HETE | 5(S)-HETE	1.87
				15(R)-HETE | (±)-8,9-EpETrE	3.43
				15(S)-HETE | 5(S)-HETE	1.87
				15(S)-HETE | (±)-8,9-EpETrE	3.43
				5(S)-HETE | (±)-8,9-EpETrE	0.84
Figure 2D	FA 20:4;O	$[M+Li]^+$	450 m/s, 20 V<br>10 passes	14R,15S-EpETrE | 15(R)-HETE	3.58
				14R,15S-EpETrE | 15(S)-HETE	3.58
				14R,15S-EpETrE | 5(S)-HETE	1.13
				14R,15S-EpETrE | (±)-8,9-EpETrE	unresolved
				15(R)-HETE | 15(S)-HETE	unresolved
				15(R)-HETE | 5(S)-HETE	1.91
				15(R)-HETE | (±)-8,9-EpETrE	3.58
				15(S)-HETE | 5(S)-HETE	1.91
				15(S)-HETE | (±)-8,9-EpETrE	3.58
				5(S)-HETE | (±)-8,9-EpETrE	1.13
Figure 2E	FA 20:4;O2	$[M+Na]^+$	400 m/s, 18 V<br>7 passes	5(S),12(S)-DiHETE | 6-trans-LTB_4	3.55
				5(S),12(S)-DiHETE | 8(S),15(S)-DiHETE	1.62
				5(S),12(S)-DiHETE | PGA_1	2.30
				6-trans-LTB_4 | 8(S),15(S)-DiHETE	2.28
				6-trans-LTB_4 | PGA_1	1.06
				8(S),15(S)-DiHETE | PGA_1	1.00
Figure 2F	FA 20:4;O2	$[M - H + CH_3COONa]^-$	400 m/s, 20 V<br>7 passes	5(S),12(S)-DiHETE | 6-trans-LTB_4	1.57
				5(S),12(S)-DiHETE | 8(S),15(S)-DiHETE	1.45
				5(S),12(S)-DiHETE | PGA_1	2.61
				6-trans-LTB_4 | 8(S),15(S)-DiHETE	3.01
				6-trans-LTB_4 | PGA_1	4.26
				8(S),15(S)-DiHETE | PGA_1	1.07
Figure S3A	FA 20:3;O3	$[M + Li]^+$	475 m/s, 25 V<br>7 passes	PGD_1 | PGE_1	0.79
				PGD_1 | $PGF_{2\alpha}$	3.34
				PGD_1 | $PGF_{2\beta}$	unresolved
				PGE_1 | $PGF_{2\alpha}$	3.26

				PGE_1 | $PGF_{2\beta}$	0.79
				$PGF_{2\alpha}$ | $PGF_{2\beta}$	3.34
Figure S3B	FA 20:4;O3	$[M + Na]^+$	400 m/s, 18 V<br>10 passes	LXA_4 | PGD_2	2.35
				LXA_4 | PGE_2	unresolved
				PGD_2 | PGE_2	2.35
Figure S3C	FA 20:4;O3	$[M - H + CH_3COONa]^-$	375 m/s, 18 V<br>7 passes	LXA_4 | PGD_2	3.69
				LXA_4 | PGE_2	4.34
				PGD_2 | PGE_2	0.61
Figure S3D	FA 20:5;O2	$[M + Li]^+$	375 m/s, 22 V<br>3 passes	PGA_2 | PGB_2	0.80
				PGA_2 | PGJ_2	4.85
				PGB_2 | PGJ_2	6.02
Figure S3E	FA 20:5;O	$[M + Li]^+$	400 m/s, 18 V<br>8 passes	5-oxo-ETE | 14(15)-EpETE	2.73
				5-oxo-ETE | 15(S)-HEPE	1.09
				5-oxo-ETE | 15-oxo-ETE	1.48
				14(15)-EpETE | 15(S)-HEPE	1.66
				14(15)-EpETE | 15-oxo-ETE	4.29
				15(S)-HEPE | 15-oxo-ETE	2.62
Figure S3F*	FA 20:4;O	$[M - H + CH_3COONa]^-$	400 m/s, 20 V<br>9 passes	15(R)-HETE | 15(S)-HETE	unresolved
				15(R)-HETE | 5(S)-HETE	1.56
				15(R)-HETE | (±)-8,9-EpETrE	1.29
				15(S)-HETE | 5(S)-HETE	1.56
				15(S)-HETE | (±)-8,9-EpETrE	1.29
				5(S)-HETE | (±)-8,9-EpETrE	2.86

* 14R,15S-EpETrE was not detected as a $[M - H + CH_3COONa]^-$ cluster under the conditions tested.

Table S3. Comparison of $^{DT}CCS_{N2}$ and $^{cIMS}CCS_{N2}$ for oxylipin standards measured across four TW conditions (15 V / 350 m/s, 18 V / 350 m/s, 18 V / 400 m/s, and 20 V / 450 m/s).

Group	Compound Abbreviation	$^{DT}CCS_{N2}$*	$^{cIMS}CCS_{N2}$ 15V and 350m/s	$^{cIMS}CCS_{N2}$ 18V and 350m/s	$^{cIMS}CCS_{N2}$ 18V and 400m/s	$^{cIMS}CCS_{N2}$ 20V and 450m/s
FA 20:3;O3	PGD_1	195.41	193.83	194.83	194.21	194.15
	PGE_1	197.54	196.19	197.29	196.47	196.46
	$PGF_{2\alpha}$	203.83	201.89	202.42	201.71	201.57
	$PGF_{2\beta}$	194.30	193.42	194.16	193.62	193.53
FA 20:4;O	14R,15S-EpETrE	184.87	184.07	184.91	184.24	184.36
	15(R)-HETE	190.00	189.10	189.89	189.49	189.39
	15(S)-HETE	190.00	188.00	189.15	188.44	188.41
	5(S)-HETE	185.78	184.46	185.22	184.84	184.95
	(±)-8,9-EpETrE	183.84	180.76	182.26	181.74	183.39
FA 20:4;O2	5(S),12(S)-DiHETE	190.56	190.48	191.80	191.06	191.18
	6-trans-LTB_4	199.00	198.35	199.57	198.80	198.90
	8(S),15(S)-DiHETE	195.42	193.72	194.65	193.99	193.87
	PGA_1	197.10	195.47	197.22	196.27	196.51
FA 20:4;O3	LXA_4	195.67	194.69	195.62	194.94	194.73
	PGD_2	192.77	190.06	191.24	190.57	190.60
	PGE_2	196.07	193.52	195.23	194.22	194.35
FA 20:5;O	14(15)-EpETE	181.62	180.14	180.88	180.51	180.52
	15(S)-HEPE	183.71	182.68	183.90	183.32	183.44
	15-oxo-ETE	190.33	189.37	190.54	189.62	189.62
	5-oxo-ETE	186.53	185.19	186.37	185.62	185.91
FA 20:5;O2	PGA_2	195.57	194.66	196.18	195.35	195.59
	PGB_2	205.39	203.79	204.49	203.73	203.39
	PGJ_2	187.73	185.62	186.60	185.84	185.82

* $^{DT}CCS_{N2}$ values were compared against Moran-Garrido et al. except where unavailable, in which case values were obtain from Silva et al.: $PGF_{2\beta}$, PGA_1, PGA_2 and PGJ_2

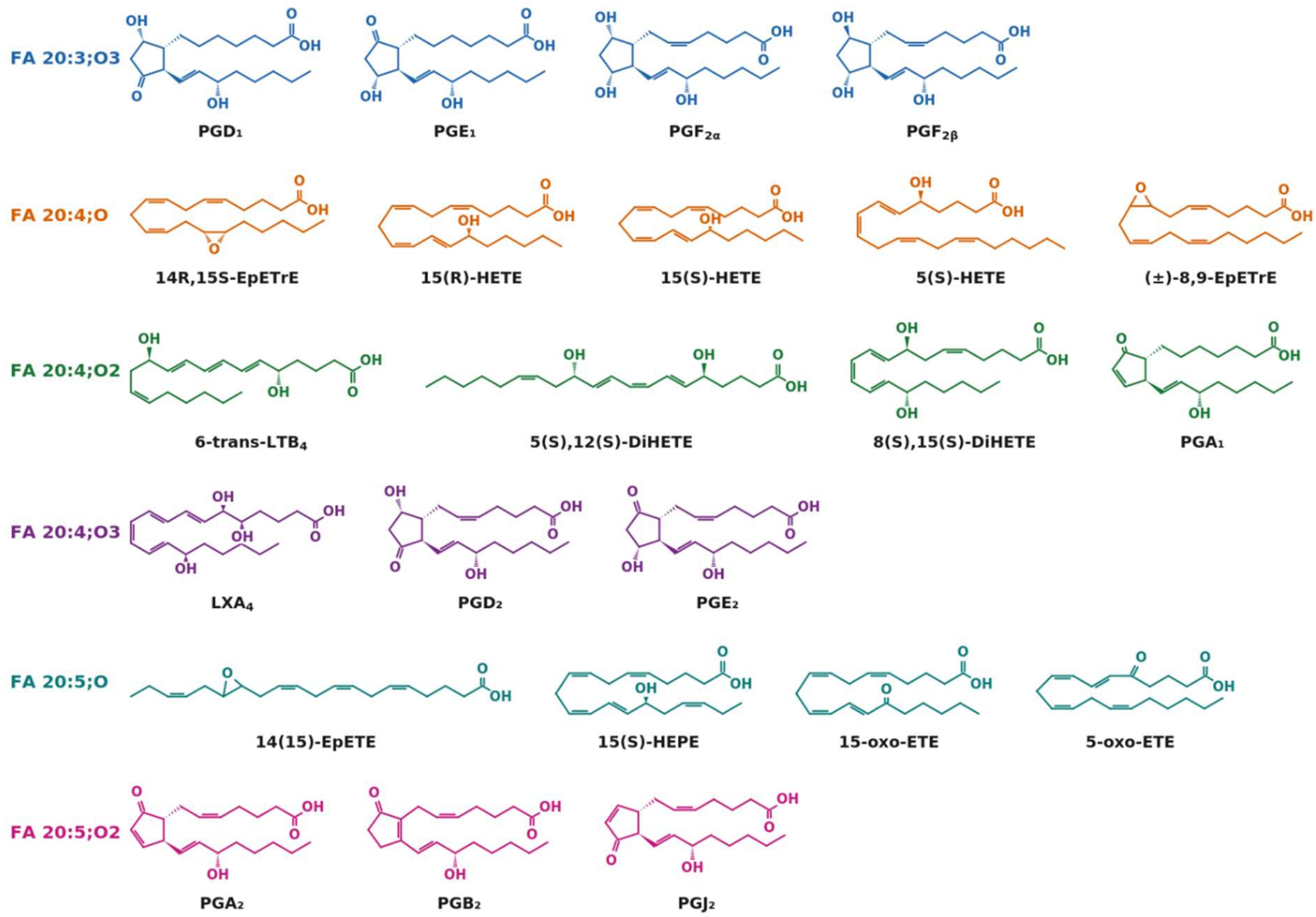

Figure S1. Chemical structures of the 23 oxylipin standards, grouped and color-coded by sum composition (FA 20:3;O3, FA 20:4;O, FA 20:4;O2, FA 20:4;O3, FA 20:5;O, and FA 20:5;O2).

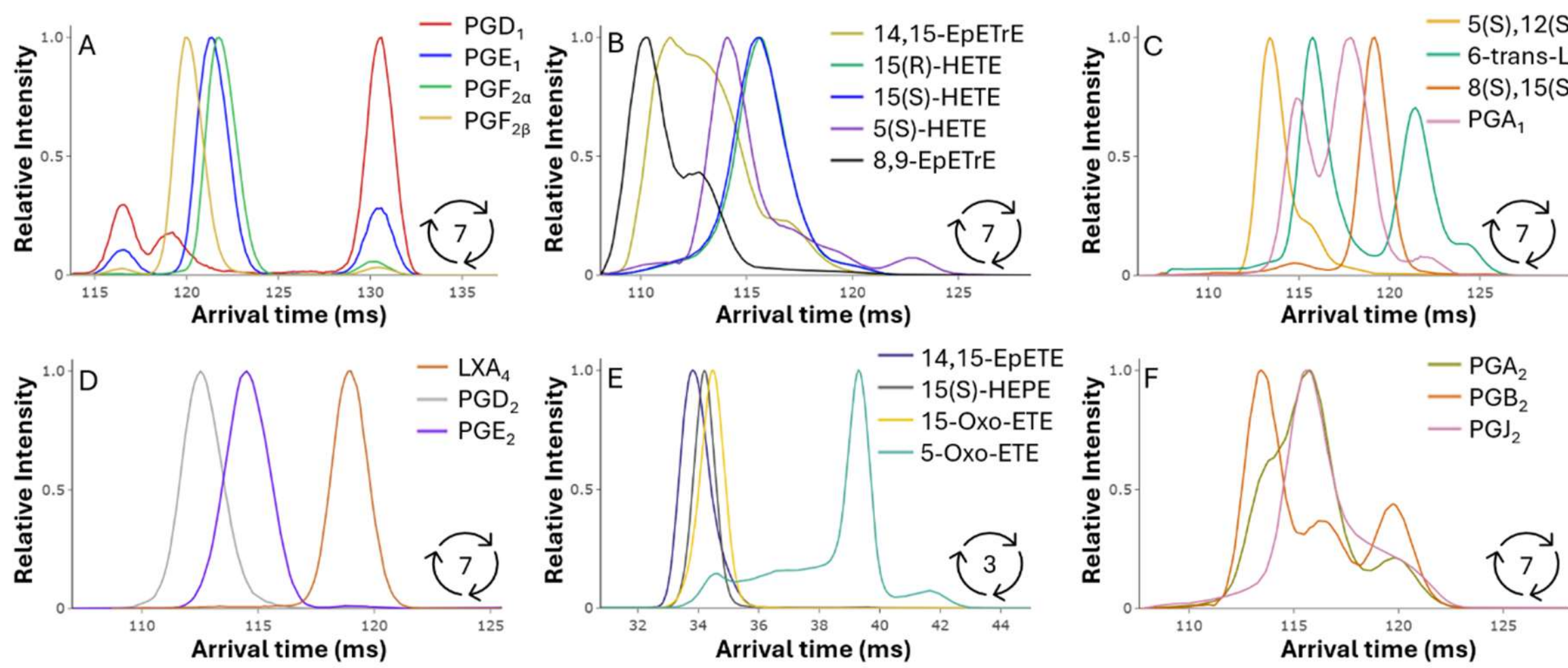

Figure S2. cIMS–MS separation of oxylipin isomeric mixtures FA 20:3;O3 (A), FA 20:4;O (B), FA 20:4;O2 (C), FA 20:4;O3 (D), FA 20:5;O (E), and FA 20:5;O2 (F) (mixtures 1-6 respectively) as their $[M - H]^-$ ions at TW conditions between 350–400 m/s and 15–18 V. Separations were extended to 7 m, except for FA 20:5;O, where 5-oxo-ETE occupied the full drift region by 3 m, limiting further separation. Only individual isomer standards are shown as solid-colored traces; no mixture trace is overlaid.

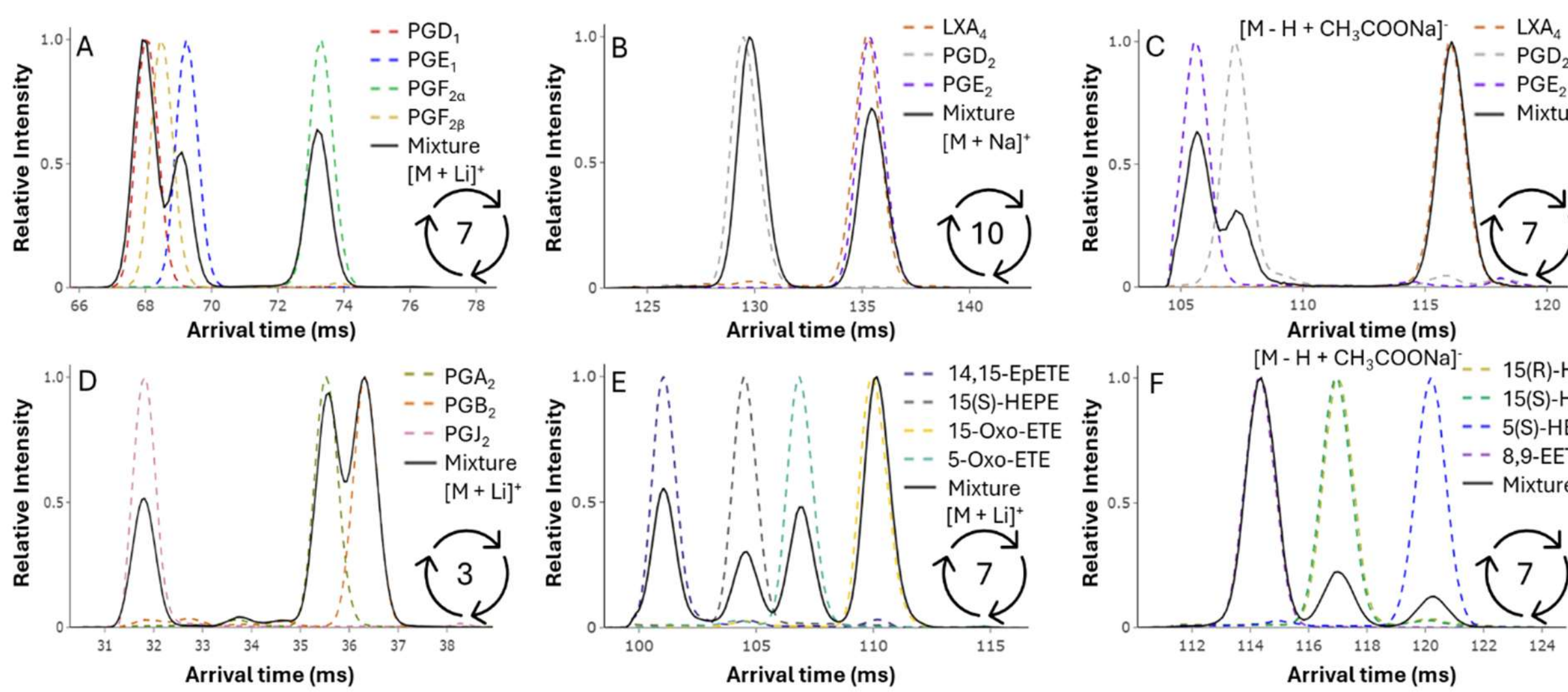

Figure S3. cIMS–MS separation of oxylipin isomeric mixtures: FA 20:3;O3 (mixture 1) $[M + Li]^+$ (A), FA 20:4;O3 (mixture 4) $[M + Na]^+$ (B) and $[M - H + CH_3COONa]^-$ (C) FA 20:5;O2 (mixture 6) $[M + Li]^+$ (D), FA 20:5;O (mixture 5) $[M + Li]^+$ (E), and FA 20:4;O (mixture 2) $[M - H + CH_3COONa]^-$ (F). Solid black traces represent isomeric mixtures, whereas dashed traces correspond to individual isomer standards analyzed separately. Optimized TW conditions are provided in Table S2.

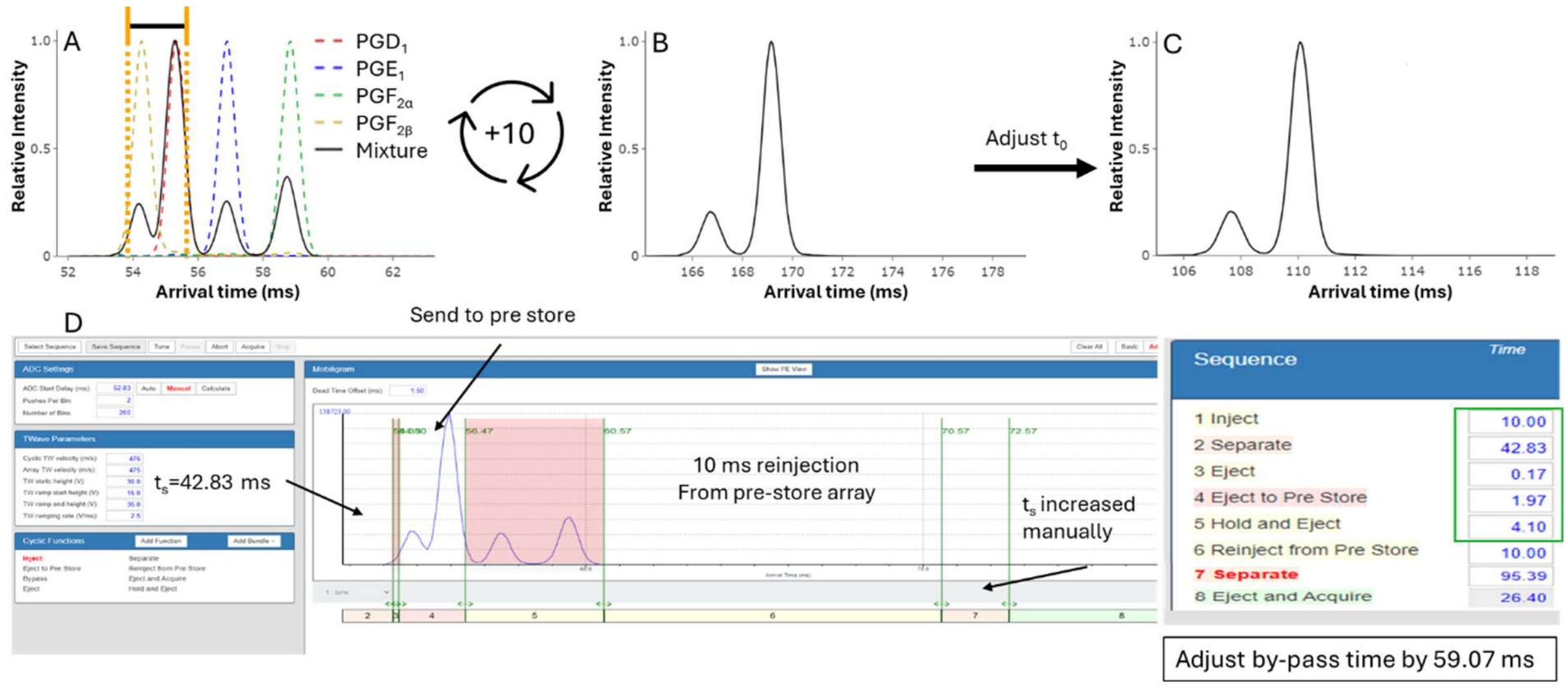

Figure S4. Multipass cIMS–MS separation of FA 20:3;O3 (mixture 1) isomers as their $[M + Na]^+$ adducts extended to the onset of ion wrap-around (A). The mobility region indicated by the gold vertical boundary was reinjected into the pre-store array and subjected to an additional 10 m of separation, resulting in an overall path length of 26 laps (8 initial passes plus 18 passes following reinjection) corresponding to 18 m of resolution due to remixing in the pre-store array (B); arrival times were subsequently corrected to account for remixing within the pre-store array (C). The experimental sequence, including the initial multipass separation (steps 1–2), transfer to the pre-store array (step 4), ejection of ions outside the boundary (steps 3 and 5), and the 18-pass separation of the selected ion packet (steps 6–8) (D). This process was repeated for the blue (PGE_1) and green ($PGF_{2\alpha}$) traces (Figure 3), and times in the final reconstructed ATD were adjusted by the sum of steps 1–5.

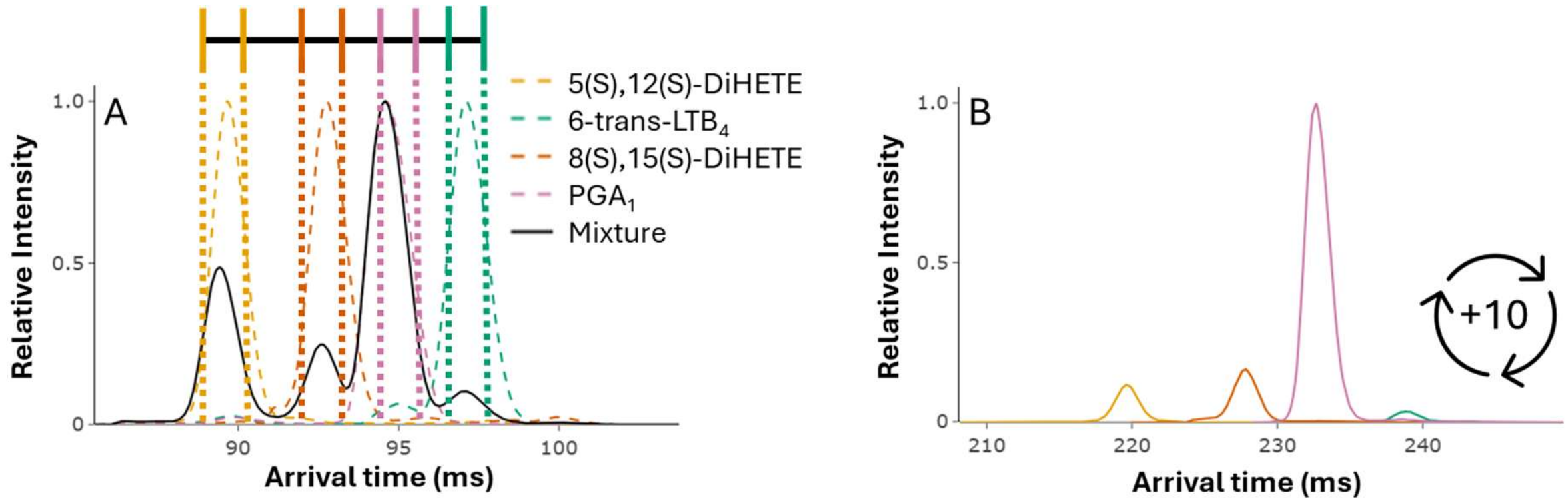

Figure S5. Multipass cIMS–MS separation of FA 20:4;O2 (mixture 3) isomers as their [M + Na]$^+$ adducts. A 7 m separation was performed at TW conditions of 400 m/s and 18 V (A). Four mobility regions from the mixture, indicated by vertical dashed boundaries, were isolated and subjected to an additional 10 m of separation, resulting in a reconstructed ATD with resolution corresponding to a cIMS pathlength of 17 m (B). Solid traces represent isomeric mixtures, and dashed traces correspond to individual isomer standards analyzed separately.

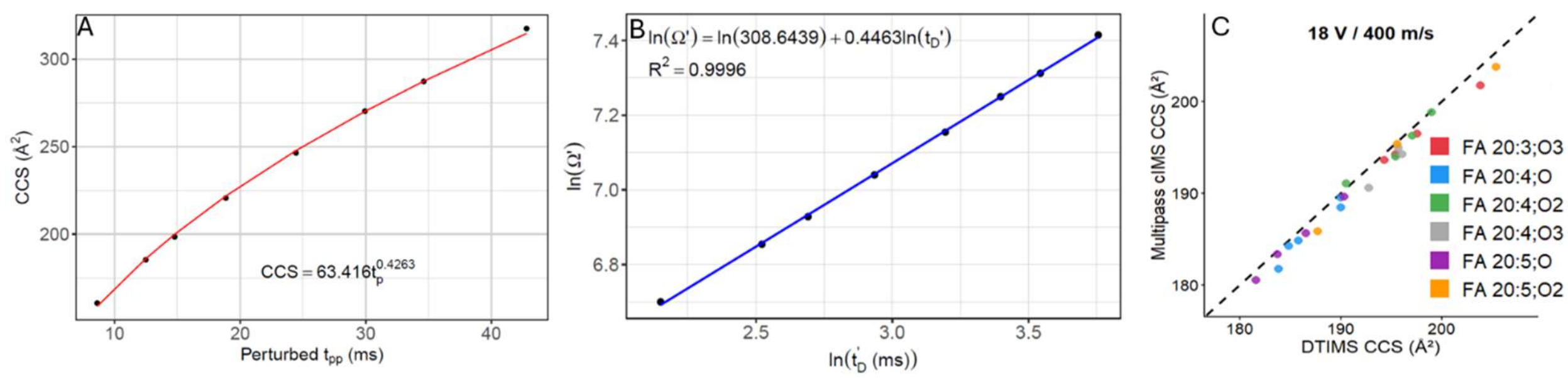

Figure S6. Multipass CCS calibration workflow for oxylipin standards at TW conditions of 18 V and 400 m/s. Calibration curve generated from t_{pp} values of [M + Na]$^+$ adducts of SpheriCal® IM–MS Low Range standards (A). Linear regression of ln(Ω′) versus ln(t_D′) used to determine the exponential factor (B). Comparison of $^{DT}CCS_{N2}$ and multipass $^{cIMS}CCS_{N2}$ for 23 oxylipin standards as [M + Na]$^+$ adducts (C).

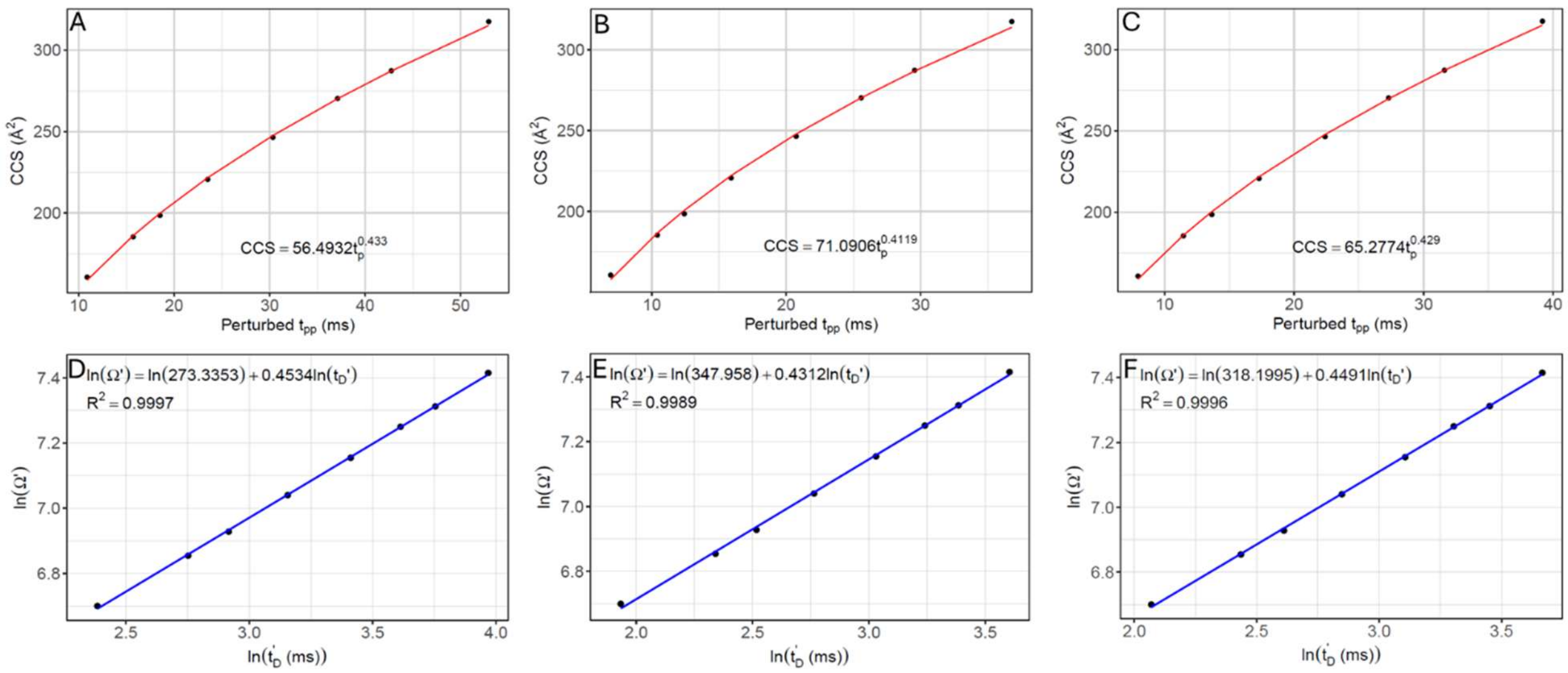

Figure S7. Multipass CCS calibration plots for all 23 oxylipin standards across representative optimized TW conditions. Power-law calibration curves generated from t_{pp} values at 15 V / 350 m/s (A), 18 V / 350 m/s (B), and 20 V / 450 m/s (C). Corresponding linear regressions of $\ln(\Omega')$ versus $\ln(t_D')$ were used to determine the exponential factor at the same TW conditions (D–F).
